# Supplementary material for: Is there a weekend effect in emergency surgery for colorectal carcinoma? Analysis from the German StuDoQ registry
Source: PLoS One. 2022 Nov 3;17(11):e0277050. doi: 10.1371/journal.pone.0277050 (PMC9632786; doi:10.1371/journal.pone.0277050)
Supplement: S1 Table — (DOCX) [file pone.0277050.s001.docx]

**Table 1 (supplementary): Basic patient characteristics**

| Variable | n=1174 |
| --- | --- |
| Age, mean±SD | 71.09±11.58 |
| Sex  Male, n (%)  Female, n (%) | 625 (53.24)  549 (46.76) |
| BMI [kg/m^2^], mean±SD | 25.79±4.98 |
| ASA, n (%)  1  2  3  4  5 | 88 (7.50)  462 (39.35)  518 (44.12)  101 (8.60)  5 (0.43) |
| Functional status, n (%)  Independent  Partially dependent  Totally dependent | 950 (80.92)  184 (15.67)  40 (3.41) |
| Comorbidities, n (%)  Arterial hypertonia  Coronary artery disease  Heart failure (NYHA I-IV)  Diabetes   - NIDDM - IDDM   History of severe COPD  Chronic steroid use  Dialysis  Disseminated cancer  Weight loss (>10% bw)  Alcohol abuse  Liver cirrhosis | 670 (57.07)  228 (19.42)  282 (24.02)  145 (12.35)  76 (6.47)  68 (5.79)  23 (1.96)  9 (0.77)  150 (12.78)  193 (16.45)  70 (5.96)  28 (2.39) |
| Surgical approach, n (%)  Open  Laparoscopic  Conversion | 985 (83.90)  81 (6.90)  108 (9.20) |
| Total operation time [min], mean±SD | 156.19±60.78 |
| Site of CRC, n (%)  Appendix vermiformis  ascending colon (+flexure)  Transverse colon  Descending colon (+flexure)  C.sigmoideum  Upper rectum (12-16cm) | 20 (1.70)  262 (22.32)  119 (10.14)  140 (11.93)  266 (22.66)  88 (7.50) |
| Resection strategy, n (%)  Right Hemicolectomy  Left Hemicolectomy  Extended Hemicolectomy  Anterior rectum resection | 625 (53.24)  299 (25.47)  139 (11.84)  111 (9.45) |
| Anastomosis, n (%)  Hand-sewn  Stapler | 502 (42.76)  672 (57.24) |
| Postoperative Length of stay [days], mean±SD  MTL30, n (%)  30-day mortality, n (%) | 17.14±11.71  220 (18.77)  71 (6.05) |
