# Supplementary material for: Is there a weekend effect in emergency surgery for colorectal carcinoma? Analysis from the German StuDoQ registry
Source: PLoS One. 2022 Nov 3;17(11):e0277050. doi: 10.1371/journal.pone.0277050 (PMC9632786; doi:10.1371/journal.pone.0277050)
Supplement: Attachment — Submitted filename: Response to reviewers comments.docx [file pone.0277050.s002.docx]

We would like to thank the reviewers for their valuable time and useful contributions. We highly appreciate the inputs you have given which definitely helped to improve our manuscript. In the following, please find point-to-point answers to your comments. All corrections or implementations are highlighted in the revised manuscript.

Reviewer #1:

1) In Results: The conversion rate in Open surgery should be specified in the text.

Thank you for this important point. We added the significant difference in the surgical approach to the results section in the text.

2) In tab 1 and 2 it is clear that in all cases an anastomosis was performed. In table 2 (now table 1) the rate of diverting stoma is specified; it would be interesting to know the indications for protective stomas.

Thank you for this query. Unfortunately, the StuDoQ registry does not provide data on the indications for protective stomas. But the most reasonable indications for diverting stoma prior surgery is a tumor stenosis, simultaneously to resection surgery is protection of the actual anastomosis. In case of a diverting stoma placement after resection surgery, patients underwent re-operation due to anastomotic leakage.

3) In the tab 2 the part related to ostomy should be better clarified: prior? After resection surgery: Is this a redo surgery due to complication?

Thank you for this valid point. We restructured this part in table 2 (now table 1) to clarify this information for the reader. It now says whether patients received a diverting stoma and at what time with regard to the resection surgery. Although the registry does not provide information on the indication for diverting stoma placement, all patients who received a diverting stoma after resection surgery had re-operation performed.

Reviewer #2:

1) There is one area of the results/discussion where I would like further clarification- Overall there was a higher rate of severe post operative complications and return to theatre for weekday versus weekend patients, but there was no difference in mortality or other outcomes. The reason for this may be due to case mix. The authors allude to the fact that defunctioning stoma is widely used in Germany for emergency cases.

We appreciate this relevant comment. Indeed, severe post-operative complications and return to theatre for weekday patients occurred (significantly) more often, but the same was found for re-admission, length of stay, MTL30 and 30d mortality. The latter remain without statistical significance, most likely due to the rather small sample size of weekend surgeries included. The higher rate of postoperative complications triggering reoperations can be explained by more extended colectomies and rectal resections performed on a weekday. This might be due to the fact that in case of obstructing colorectal cancer the sole creation of an ostomy without resection surgery is performed more often on a weekend. However, patients without resection of their colorectal cancer were not included in this study. Case mix seems to be a rather unlikely reason for a higher rate of postoperative complications and reoperations for weekday patients, since UICC tumor stages in patients who underwent emergency colorectal cancer resections on the weekend were significantly higher than in patients who received emergency surgery on a weekday. In addition, no difference was found for Age, BMI, ASA, functional status and comorbidities between the two groups (new table 1).

2) It would be helpful if the authors could explicitly state (or show on a table) whether there is any significant difference in the type of surgery performed on different days. For example were there more resectional operations done on Mon-Fri and more defunctioning operations done on a weekend?

Thank you for this important query. There is a significant difference in resection strategy between weekday and weekend surgery, mostly due to more rectum resections on weekdays (10.4%) compared to weekends (2.8%) (new table 1). This led to the conclusion, that emergencies due to e.g. obstructing rectal cancer were managed by decompressing ostomy as bridge to surgery, as pointed out in the discussion section (second paragraph). Cases with no resection but just decompressing or defunctioning surgery in terms of stoma creation were not included in this study. Regarding the creation of a diverting stoma, no difference could be found between weekday and weekend emergency resection surgeries due to colorectal cancer.

3) There are also several areas where the grammar/ syntax should be changed-

Please excuse us for this shortcoming and thank you for the detailed revision. We changed all errors according to your suggestions.
